# Metformin Protects Against Radiation-Induced Heart Injury and Attenuates the Upregulation of Dual Oxidase Genes Following Rat’s Chest Irradiation

**DOI:** 10.22088/IJMCM.BUMS.7.3.193

**Published:** 2018-10-21

**Authors:** Rasoul Yahyapour, Peyman Amini, Hana Saffar, Saeed Rezapoor, Elahe Motevaseli, Mohsen Cheki, Bagher Farhood, Farzad Nouruzi, Dheyauldeen Shabeeb, Ahmed Eleojo Musa, Masoud Najafi

**Affiliations:** 1 *School of Medicine, Jiroft University of Medical Sciences, Jiroft, Iran.*; 2 *Department of Radiology, Faculty of Paramedical, Tehran University of Medical Sciences, Tehran, Iran.*; 3 *Imam Khomeini Hospital Complex, Tehran University of Medical Sciences, Tehran, Iran.*; 4 *Department of Molecular Medicine, School of Advanced Technologies in Medicine, Tehran University of Medical Sciences, Tehran, Iran.*; 5 *Department of Radiologic Technology, Faculty of Paramedicine, Ahvaz Jundishapur University of Medical Sciences, Ahvaz, Iran.*; 6 *Departments of Medical Physics and Radiology, Faculty of Paramedical Sciences, Kashan University of Medical Sciences, Kashan, Iran.*; 7 *Department of Medical Radiation Engineering, Science and Research Branch, Islamic Azad University, Tehran, Iran.*; 8 *Department of Medical Physics and Biomedical Engineering, Faculty of Medicine, Tehran University of Medical Sciences (International Campus), Tehran, Iran.*; 9 *Department of Physiology, College of Medicine, University of Misan, Misan, Iraq.*; 10 *Research Center for Molecular and Cellular Imaging, Tehran University of Medical Sciences (International Campus), Tehran, Iran.*; 11 *Radiology and Nuclear Medicine Department, School of Paramedical Sciences, Kermanshah University of Medical Sciences, Kermanshah, Iran.*

**Keywords:** Radiation, Metformin, Heart Injury, IL-4, IL-13; DUOX1, DUOX2

## Abstract

Radiation-induced heart toxicity is one of the serious side effects after a radiation disaster or radiotherapy for patients with chest cancers, leading to a reduction in the quality of life of the patients. Evidence has shown that infiltration of inflammatory cells plays a key role in the development of functional damages to the heart via chronic upregulation of some pro-fibrotic and pro-inflammatory cytokines. These changes are associated with continuous free radical production and increased stiffness of heart muscle. IL-4 and IL-13 are two important pro-fibrotic cytokines which contribute to the side effects of ionizing radiation exposure. Recent studies have proposed that IL-4 through upregulation of *DUOX2*, and IL-13 via stimulation of *DUOX1* gene expression, are involved in the development of radiation late effects. In the present study, we aimed to detect changes in the expression of these pathways following irradiation of rat’s heart. Furthermore, we evaluated the possible protective effect of metformin on the development of these abnormal changes. 20 male rats were divided into 4 groups (control, radiation, metformin treated, metformin + radiation). These rats were irradiated with 15 Gy ^60^Co gamma rays, and sacrificed after 10 weeks for evaluation of the changes in the expression of *IL4R1*, *IL-13R2a*, *DUOX1* and *DUOX2*. In addition, the levels of IL-4 and IL-13 cytokines, as well as infiltration of macrophages and lymphocytes were detected. Results showed an upregulation of both *DUOX1* and *DUOX2 *pathways in the presence of metformin, while the level of IL-13 did not show any significant change. This was associated with infiltration of macrophages and lymphocytes. Also, treatment with metformin could significantly attenuate accumulation of inflammatory cells, and upregulate these pathways. Therefore, **s**uppression of dual oxidase genes by metformin may be a contributory factor to its protective effect.

Radiotherapy is a non‐invasive cancer treatment modality prescribed for more than half of patients with solid tumors during their treatment course. However, acute and late detrimental effects of exposure to radiation affect the deliverable intensity of radiotherapy (1). In addition, these side effects can reduce the quality of life of cancer patients. Radiation-induced heart damage is one of the serious side effects after radiotherapy of lung and breast cancers (2, 3). Several studies have shown that in addition to the beneficial effect of radiotherapy in reducing local recurrences, there is some evidence showing that overall survival is hampered by an increased risk of non-cancerous diseases such as heart disease (4). Radiation-induced late cardiac damages such as coronary and carotid arteries diseases, ischemic heart disease etc., have been known for some decades (5). Earlier studies showed a high risk of heart diseases for women with left sided breast cancer who underwent radiotherapy (6). An increased risk of myocardial infarction (MI) for left breast cancer has been observed in comparison with the right side (7, 8).

So far, several experiments have been conducted to detect the mechanisms involved in radiation-induced heart diseases. Amongst the various factors, fibrosis and inflammation play a key role (9). The long term upregulation of some cytokines such as interleukin 1 (IL-1), IL-4, IL-13, tumor necrosis factor alpha (TNF-α) and transforming growth factor beta (TGF-β) have pivotal roles in the development of radiation-induced fibrosis (10). IL-4 and IL-13 are two important factors that through stimulation of reactive oxygen species (ROS) production promote the production of collagen and extracellular matrix, resulting in stiffness of normal tissue (11, 12). This effect causes damage to the normal function of tissues, especially in the lung, heart, and gastrointestinal organs (13).

Although, advancements in radiotherapy techniques can improve the management of side effects, several studies have proposed some radioprotective agents for ameliorating long-term consequences. IL-4 is a key cytokine involved in the late effects of radiation especially fibrosis, and is mainly released by macrophages (14). In addition to its direct role in the late effects of radiation, it can stimulate the regulation of other pro-fibrotic and pro-inflammatory cytokines such as TGF-β and IL-13 (15). These cytokines stimulate the production of free radicals for a long period which mediates chronic oxidative injury and collagen deposition in intracellular space (16). Moreover, increased level of these cytokines can promote reduction/oxidation interaction, and stimulate carcinogenesis (17). Targeting IL-4 and IL-13 is an interesting idea for suppressing the development of fibrosis after radiotherapy (18). However, amelioration of oxidative damage by some agents such as natural antioxidants, herbal compounds, and anti-inflammatory agents have shown promising results (19, 20).

Metformin is an anti-diabetic drug which has shown some antioxidant, anti-fibrotic, and radioprotective effects (21, 22). Studies proposed that these effects of metformin are a result of suppression of some ROS mediator genes such as ubiquinone oxidoreductase, NADPH oxidase 4 (NOX4), and stimulation of antioxidant enzymes (23-25). In this study, we evaluated the protective effect of metformin on radiation-induced heart injury, and regulation of IL-4 and IL-13 signaling pathways.

## Materials and methods

**Experimental design**

20 male rats were randomly divided into 4 groups. Group 1: 5 rats were selected as controls without any intervention except intraperitoneal injection of ketamine and xylazine similar to other groups. Group 2: 5 rats received 100 mg/kg dose of metformin for 4 and 5 days before and after exposure to 15 Gy γ-rays, respectively. Metformin was administered 30 min before irradiation. Group 3: 5 rats were treated with metformin for 10 days. Group 4: 5 rats received 15 Gy γ-rays to the heart without metformin treatment. At 10 weeks after irradiation, all rats were sacrificed and their heart tissues were extracted from the chest. Afterwards, both ventricles were fixed in 10% buffered formalin and auricles were frozen at -80^ o^C.

**Administration of metformin**

Metformin was purchased from Tehran Chemi Company, Tehran, Iran. It was dissolved in distilled water (20mg/ml) and administered to rats (1ml per day) orally for 9 consecutive days (4 and 5 days before and after irradiation respectively). On the day of irradiation, metformin was administered 30 min before commencement. 100 mg/kg of metformin was selected as a non-toxic dose based on a previous study (26).

**Irradiation of animals**

Before irradiation, all rats were anesthetized with an intraperitoneal injection of ketamine and xylazine at doses of 80 and 5 mg/kg, respectively. In two groups, rats were irradiated locally on the chest area with a single dose of 15 Gy γ-rays and a radiation field of 6×6 cm^2^. This dose of radiation was selected according to previous studies for inducing heart injury (27). The source-to-skin distance was 60 cm with a dose rate of 109 cGy/min.

**Gene expression analysis**

Total RNA was isolated from homogenates of auricles using TRIzol reagent (Sina gene, Iran). Afterwards, cDNA was synthetized using the reverse transcription kit (Takara, Japan). PCR reactions were performed in a volume of 10 µl containing 5 µl SYBR Green master mix (Takara, Japan). The expression of each gene was detected using real-time PCR with *PGM1* as internal control. Primers for each gene are shown in table 1. Real-Time PCR efficiency for all genes was determined using the slope of a linear regression which described by Pfaffl (28). 5 samples in each group were run in duplicate.

**Table 1:** The sequence of primers for Real-time PCR

***Gene ***	***Forward sequence ***	***Reverse sequence***
***IL-4R1***	GAGTGAGTGGAGTCCCAGCATC	GCTGAAGTAACAGGTCAGGC
***IL-13Ra2***	TCGTGTTAGCGGATGGGGAT	GCCTGGAAGCCTGGATCTCTA
***DUOX1***	AAGAAAGGAAGCATCAACACCC	ACCAGGGCAGTCAGGAAGAT
***DUOX2***	AGTCTCATTCCTCACCCGGA	GTAACACACACGATGTGGCG
***PGM1***	CATGATTCTGGGCAAGCACG	GCCAGTTGGGGTCTCATACAAA

**Fig. 1:**
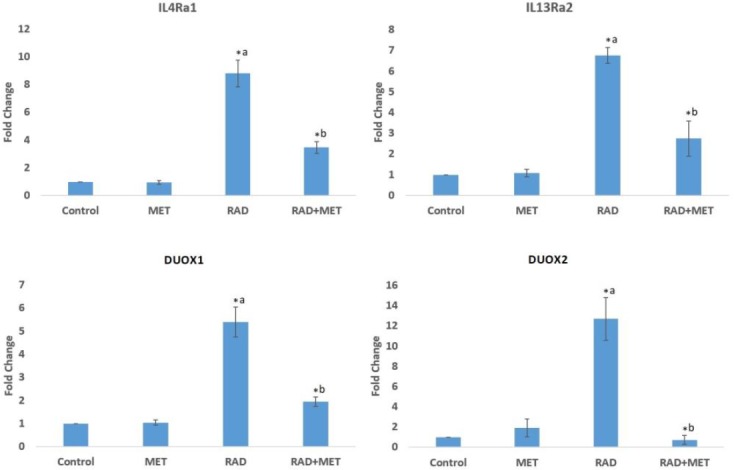
The expression of IL4Ra1, IL13Ra2, DUOX1, and DUOX2 in different groups. Results show an increase in the expression of genes following exposure to radiation. Treatment with metformin alleviates upregulation of these genes (t-test, P < 0.05, a=significant compared to control; b: significant compared to radiation group; MET=metformin treatment; RAD= Radiation group; RAD+MET= radiation plus metformin treatment)

**Fig. 2:**
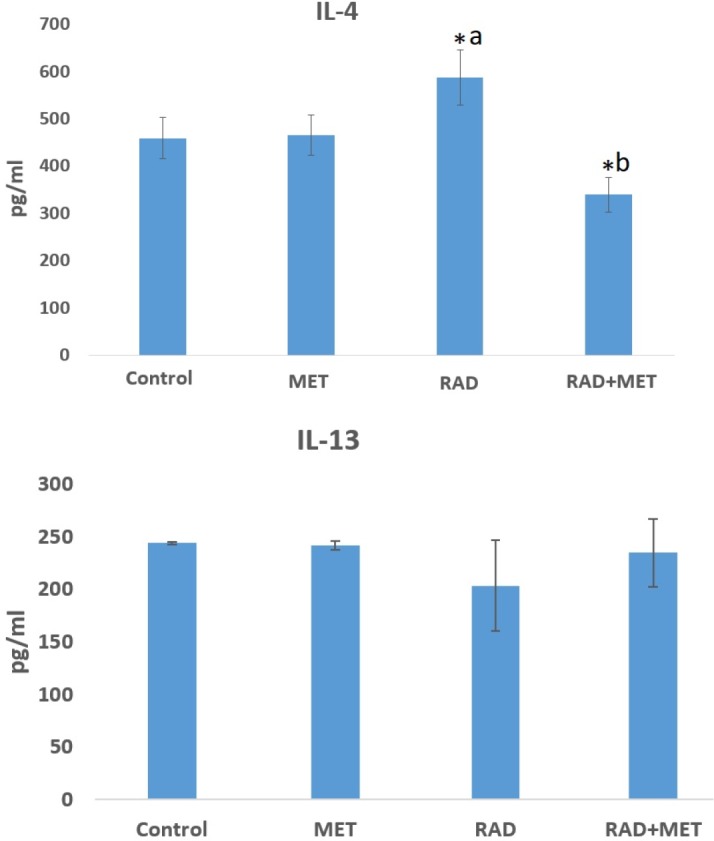
The levels of IL-4 and IL-13 in rat’s heart tissues following irradiation or treatment with metformin before irradiation. Irradiation caused a significant increase in IL-4, while treatment with metformin before irradiation decreased its level. Data also did not show any significant change in the level of IL-13 (t-test, P < 0.05, a=significant compared to control; b: significant compared to radiation group; MET= metformin treatment; RAD= Radiation group; RAD+MET= radiation plus metformin treatment)

**Fig. 3:**
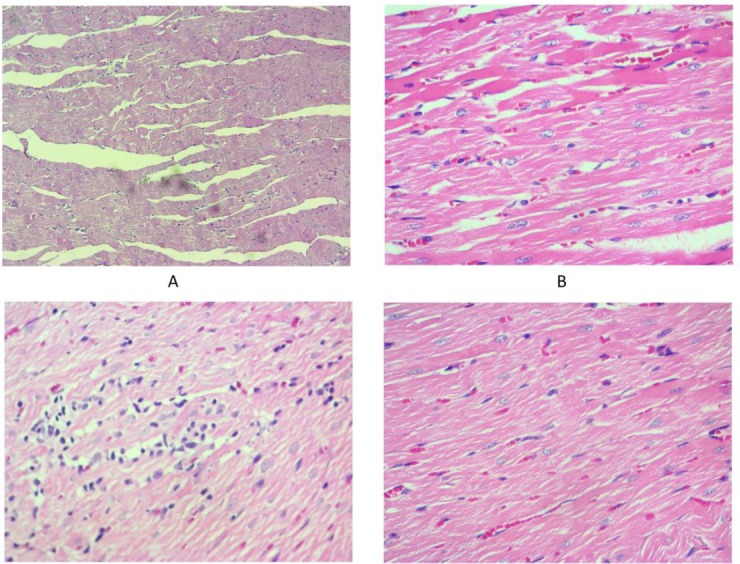
Infiltration of inflammatory cells (mainly lymphocytes) in the heart tissues following irradiation of rat’s chest. Infiltration of these cells in radiation + metformin group was similar to control group. A: Control; B: metformin; C: radiation; D: radiation + metformin (H&E staining)

**Evaluation of IL-4 and IL-3 levels**

The levels of both IL-4 and IL-13 were detected using ELISA kits (Zellbio ELISA kits, Germany) based on the manufacturer's protocol. 

**Histopathological evaluation**

After fixation, the heart tissues were embedded in paraffin. Sections of the ventricles were cut at 5 µm, and then stained with hematoxylin and eosin (H&E). The obtained sections were evaluated for infiltration of macrophages and lymphocytes. All histopathological studies were performed at the Imam Khomeini Hospital, Tehran University of Medical Sciences, Tehran, Iran. The blinded evaluation was performed using a light microscope.

**Statistical analysis**

Data were analyzed using SPSS software version 16 (IBM, Chicago, USA). Real-time PCR results were analyzed by T-Test. ELISA results were analyzed by ANOVA test (Tukey’s HSD post hoc). Histopathological results were analyzed using Mann–Whitney. A P value <0.05 was considered as statistically significant.

## Results

**Gene expression analysis**

Irradiation of rat’s chest caused the upregulation of *IL-4Ra1* (8.80±0.97 fold) in comparison with the control group (P= 0.009). Treatment with metformin led to a significant reduction in *IL-4Ra1* in comparison with radiation group (3.47±0.41 fold, P= 0.006). Results showed a significant upregulation of *IL13Ra2* (6.76±0.39 fold, P < 0.001) compared to control, while its expression was attenuated when rats were treated with metformin (2.75±0.85 fold, P < 0.001). The expression of *DUOX1* showed an increase following irradiation (5.40±0.65 fold, P < 0.001), however, its expression droped when treatment was performed using metformin (1.94±0.20 fold, P< 0.001). We also observed that the exposure to irradiation caused a significant increase in the expression of *DUOX2* in comparison with the control (12.69±2.09 fold, P= 0.01). Treatment with metformin resulted in potent decrease in *DUOX2* expression (0.73±0.45 fold, P= 0.016) (Figure 1).

**Evaluation of IL-4 and IL-3 levels**

Treatment with metformin alone did not cause any significant change in the level of IL-4. However, exposure to irradiation led to a significant increase of IL-4 in comparison with the control group (640±53 vs 413±37) (P=0.002). With metformin treatment before irradiation, the level of IL-4 decreased potently (339±44, p<0.001). In addition, ELISA results showed no significant change in the level of IL-13 (244.15±1.37 vs 203.56±43.39) (Figure 2).

**Macrophages and lymphocytes infiltration**

Histopathological evaluation showed that irradiation caused a mild increase in the infiltration of inflammatory cells, including macrophages and lymphocytes. This was more obvious for lymphocytes. However, treatment with metformin completely reversed the infiltration of inflammatory cells (Figure 3).

## Discussion

Radiation- induced heart damage is one of the most threatening non- cancerous diseases following exposure to irradiation. Heart injury and subsequent diseases have been detected following whole body or partial body exposure to irradiation during a radiation disaster or radiotherapy (29). Incidence of heart diseases is one of the most common reasons of increased mortality among people who were exposed to irradiation during Hiroshima and Nagasaki atomic bomb explosion (30). Similarly, elevated heart diseases have been confirmed among the survivors of the Chernobyl disaster (30). This issue is more pronounced in patients with left breast cancer or lung cancer, when the heart is located within the radiation field (32). Evidence has proposed that increased cardiovascular disease such as carotid and coronary artery fibrosis, pericarditis, ischemia and hypertrophy are mostly common after exposure to irradiation. Histological studies have shown that accumulation of inflammatory cells such as mast cells, macrophages, and lymphocytes play a key role in chronic oxidative damage, inflammation and fibrosis, leading to changes in the normal structure of heart tissue and increased risk of heart attack (33).

It has been confirmed that accumulation of inflammatory cells following exposure to irradiation mediates normal tissue destruction through various signaling pathways. Increased levels of IL-1, IL-4, IL-6, IL-8, IL-13, IL-33, TGF-β, and TNF-α have been observed in several studies (1). In this study, we aimed to evaluate whether or not IL-4–IL4Ra1 and IL-13–IL13Ra2 signaling pathways are increased following exposure of the heart to irradiation. A previous study by Hassani et al. showed that IL-4 through *DUOX2* and IL-13 through *DUOX1* promote chronic ROS production in thyroid tumor cells (34). As shown in figure 3, exposure of rat’s heart to radiation led to significant infiltration of macrophages and lymphocytes. Figure 2 shows a significant increase in the level of IL-4, but not IL-13. We also evaluated the expression of downstream genes, including *IL-4Ra1*, *IL13Ra2*, *DUOX1*, and *DUOX2*. Results indicated that exposure to radiation led to a significant upregulation of all examined genes. Hence, both DUOX1 and DUOX2 are potential pathways that are upregulated following irradiation of the heart, and may be involved in radiation toxicity. Treatment with metformin caused potent attenuation of these genes associated with amelioration of infiltration of macrophages and lymphocytes. Probably, upregulation of *DUOX1* and *IL13Ra2* are mediated through other signaling pathways. It has been confirmed that in addition to *IL-13*, *DUOX1* and *IL13Ra2* can be upregulated through IL-4 (35). There is a possibility that IL-4 upregulates *DUOX1* and *DUOX2* genes expression through stimulation of IL4Ra1, while IL-13 and IL-13Ra2 has no effect on this pathway. Moreover, some other cytokines such as interferon gamma (IFN-γ) have shown stimulatory effects on the expression of these genes (34, 36).

So far, some agents have been proposed for protection against heart injuries due to irradiation. In our previous study, we showed that hesperidin as a natural agent has the ability to alleviate infiltration of immune cells including macrophages, lymphocytes and mast cells. This was associated with alleviation of collagen accumulation, and decreased oxidative stress (37). Gurses et al. evaluated the radioprotective effect of amifostine in rat’s heart tissues. They showed that a 200 mg/kg dose was able to alleviate necrosis and vascular damage in heart cells, while it could not prevent fibrosis in epicardial and myocardial tissues (38). In another study, they showed that melatonin can attenuate late effects of irradiation such as fibrosis, vasculitis, and necrosis in rat’s heart (39).

In recent years, some studies have been conducted to evaluate the radioprotective effects of metformin in both *in vitro* and *in vivo* studies. In an *in vitro* study, Cheki et al. showed that pre-treatment with metformin can reduce the formation of micronuclei in human lymphocytes (40) while an *in vivo* study by Xu et al. showed that metformin via down-regulation of NADPH oxidase 4 (*NOX4*) reduces chronic ROS production in the hematopoietic stem cells of mice (22). Miller et al. showed that treatment of mice with metformin before or even 24 h after exposure to irradiation can mitigate lethal effects of radiation by a protection factor of 1.8 (26). In the present study, our results propose that metformin treatment can attenuate the upregulation of dual oxidase genes in rat’s heart following exposure to ionizing radiation. Although, the exact mechanisms for radioprotective effect of metformin remain to be elucidated, it seems that antioxidant and anti-inflammatory effects play an important role (41). Also, through stimulation of 5' adenosine monophosphate-activated protein kinase (AMPK), metformin is able to induce DNA repair, leading to the reduction of accumulated DNA damage and cell death (42, 43). Antioxidant property of metformin that is mediated through stimulation of antioxidant enzymes may reduce radiation injury via neutralization of radiation-induced ROS and DNA damage, and also modulation of inflammatory cells activity (25). Also, via enhancement of DNA repair capacity (which is critical after exposure to ionizing radiation), metformin may attenuate long term consequences of radiation (26, 44). DNA damage and cell death following exposure to radiation cause the release of several pro-inflammatory and pro-fibrotic cytokines, which mediate the upregulation of pro-oxidant enzymes (17). These changes are responsible for several side effects. Thus, metformin may through attenuation of free radical production, DNA damage response as well as cytokines release, mediate several side effects following exposure to radiation.

Metformin has shown the ability to protect the heart against toxic effects of other agents such as chemotherapy drugs. A study by Zilinyi et al. revealed the protective effect of metformin against doxorubicin cytotoxicity in rats. Results showed that treatment of rats with metformin reduces oxidative injury and ameliorates increased level of troponin (a marker of cardiac toxicity). Moreover, metformin treatment reduced the thickening of myofibrillum in heart muscles (45). Moreover, it has been shown that metformin alleviates inflammation, apoptosis induction, and histological changes in rat’s heart tissues following doxorubicin injection (46, 47).

In conclusion, the present study showed that exposing rat’s chest to irradiation caused a significant upregulation of proinflammation and pro-fibrotic genes including *IL4Ra1*, *IL-13Ra2*, *DUOX1*, and *DUOX2* in heart tissues. Moreover, infiltration of macrophages and lymphocytes increased. Treatment with metformin led to potent inhibitory effects on both histopathological changes and all mentioned genes. Our results showed that IL-13 is not involved in heart injury following exposure to irradiation.

## References

[1] Najafi M, Motevaseli E, Shirazi A (2018). Mechanisms of inflammatory responses to radiation and normal tissues toxicity: clinical implications. Int J Radiat Biol.

[2] Darby SC, Cutter DJ, Boerma M (2010). Radiation-related heart disease: current knowledge and future prospects. Int J Radiat Oncol Biol Phys.

[3] Schultz-Hector S (1992). Radiation-induced heart disease: review of experimental data on dose response and pathogenesis. Int J Radiat Biol.

[4] Little MP (2013). A review of non-cancer effects, especially circulatory and ocular diseases. Radiat Environ Biophys.

[5] Little MP (2009). Cancer and non-cancer effects in Japanese atomic bomb survivors. J Radiol Prot.

[6] Sardaro A, Petruzzelli MF, D'Errico MP (2012). Radiation-induced cardiac damage in early left breast cancer patients: risk factors, biological mechanisms, radiobiology, and dosimetric constraints. Radiother Oncol.

[7] Corradini S, Ballhausen H, Weingandt H (2018). Left-sided breast cancer and risks of secondary lung cancer and ischemic heart disease : Effects of modern radiotherapy techniques. Strahlenther Onkol.

[8] Taylor CW, Kirby AM (2015). Cardiac Side-effects From Breast Cancer Radiotherapy. Clin Oncol (R Coll Radiol).

[9] Cuomo JR, Sharma GK, Conger PD (2016). Novel concepts in radiation-induced cardiovascular disease. World J Cardiol.

[10] Kolivand S, Amini P, Saffar H (2018). Evaluating the radioprotective effect of curcumin on rat's heart tissues. Current radiopharmaceuticals.

[11] Chung SI, Horton JA, Ramalingam TR (2016). IL-13 is a therapeutic target in radiation lung injury. Sci Rep.

[12] Groves AM, Johnston CJ, Misra RS (2016). Effects of IL-4 on pulmonary fibrosis and the accumulation and phenotype of macrophage subpopulations following thoracic irradiation. Int J Radiat Biol.

[13] Hojan K, Milecki P (2014). Opportunities for rehabilitation of patients with radiation fibrosis syndrome. Rep Pract Oncol Radiother.

[14] Buttner C, Skupin A, Reimann T (1997). Local production of interleukin-4 during radiation-induced pneumonitis and pulmonary fibrosis in rats: macrophages as a prominent source of interleukin-4. Am J Respir Cell Mol Biol.

[15] Kaviratne M, Hesse M, Leusink M (2004). IL-13 activates a mechanism of tissue fibrosis that is completely TGF-beta independent. J Immunol.

[16] Lee CG, Homer RJ, Zhu Z (2001). Interleukin-13 induces tissue fibrosis by selectively stimulating and activating transforming growth factor beta(1). J Exp Med.

[17] Farhood B, Goradel NH, Mortezaee K (2018). Intercellular communications-redox interactions in radiation toxicity; potential targets for radiation mitigation. J Cell Commun Signal.

[18] Jakubzick C, Kunkel SL, Puri RK (2004). Therapeutic targeting of IL-4- and IL-13-responsive cells in pulmonary fibrosis. Immunol Res.

[19] Yahyapour R, Amini P, Rezapour S (2018). Radiation-induced inflammation and autoimmune diseases. Mil Med Res.

[20] Yahyapour R, Shabeeb D, Cheki M (2018). Radiation protection and mitigation by natural antioxidants and flavonoids; implications to radiotherapy and radiation disasters. Curr Mol Pharmacol.

[21] Birben E, Sahiner UM, Sackesen C (2012). Oxidative stress and antioxidant defense. World Allergy Organ J.

[22] Xu G, Wu H, Zhang J (2015). Metformin ameliorates ionizing irradiation-induced long-term hematopoietic stem cell injury in mice. Free Radic Biol Med.

[23] Kelly B, Tannahill GM, Murphy MP (2015). Metformin Inhibits the Production of Reactive Oxygen Species from NADH:Ubiquinone Oxidoreductase to Limit Induction of Interleukin-1beta (IL-1beta) and Boosts Interleukin-10 (IL-10) in Lipopolysaccharide (LPS)-activated Macrophages. J Biol Chem.

[24] Sato N, Takasaka N, Yoshida M (2016). Metformin attenuates lung fibrosis development via NOX4 suppression. Respir Res.

[25] Buldak L, Labuzek K, Buldak RJ (2014). Metformin affects macrophages' phenotype and improves the activity of glutathione peroxidase, superoxide dismutase, catalase and decreases malondialdehyde concentration in a partially AMPK-independent manner in LPS-stimulated human monocytes/macrophages. Pharmacol Rep.

[26] Miller RC, Murley JS, Grdina DJ (2014). Metformin exhibits radiation countermeasures efficacy when used alone or in combination with sulfhydryl containing drugs. Radiat Res.

[27] Kruse JJ, Zurcher C, Strootman EG (2001). Structural changes in the auricles of the rat heart after local ionizing irradiation. Radiother Oncol.

[28] Pfaffl MW (2001). A new mathematical model for relative quantification in real-time RT-PCR. Nucleic Acids Res.

[29] Barjaktarovic Z, Schmaltz D, Shyla A (2011). Radiation-induced signaling results in mitochondrial impairment in mouse heart at 4 weeks after exposure to X-rays. PLoS One.

[30] Shimizu Y, Kodama K, Nishi N (2010). Radiation exposure and circulatory disease risk: Hiroshima and Nagasaki atomic bomb survivor data, 1950-2003. BMJ.

[31] Ivanov VK, Maksioutov MA, Chekin S (2000). Radiation-epidemiological analysis of incidence of non-cancer diseases among the Chernobyl liquidators. Health Phys.

[32] Eldabaje R, Le DL, Huang W (2015). Radiation-associated Cardiac Injury. Anticancer Res.

[33] Taunk NK, Haffty BG, Kostis JB (2015). Radiation-induced heart disease: pathologic abnormalities and putative mechanisms. Front Oncol.

[34] Ameziane-El-Hassani R, Talbot M, de Souza Dos Santos MC (2015). NADPH oxidase DUOX1 promotes long-term persistence of oxidative stress after an exposure to irradiation. Proc Natl Acad Sci U S A.

[35] Eskalli Z, Achouri Y, Hahn S (2016). Overexpression of Interleukin-4 in the Thyroid of Transgenic Mice Upregulates the Expression of Duox1 and the Anion Transporter Pendrin. Thyroid.

[36] Harper RW, Xu C, Eiserich JP (2005). Differential regulation of dual NADPH oxidases/peroxidases, Duox1 and Duox2, by Th1 and Th2 cytokines in respiratory tract epithelium. FEBS Lett.

[37] Rezaeyan A, Haddadi GH, Hosseinzadeh M (2016). Radioprotective effects of hesperidin on oxidative damages and histopathological changes induced by X-irradiation in rats heart tissue. J Med Phys.

[38] Gurses I, Ozeren M, Serin M (2018). Histopathological efficiency of amifostine in radiationinduced heart disease in rats. Bratisl Lek Listy.

[39] Gurses I, Ozeren M, Serin M (2014). Histopathological evaluation of melatonin as a protective agent in heart injury induced by radiation in a rat model. Pathol Res Pract.

[40] Cheki M, Shirazi A, Mahmoudzadeh A (2016). The radioprotective effect of metformin against cytotoxicity and genotoxicity induced by ionizing radiation in cultured human blood lymphocytes. Mutat Res.

[41] Hirsch HA, Iliopoulos D, Struhl K (2013). Metformin inhibits the inflammatory response associated with cellular transformation and cancer stem cell growth. Proc Natl Acad Sci U S A.

[42] Algire C, Moiseeva O, Deschenes-Simard X (2012). Metformin reduces endogenous reactive oxygen species and associated DNA damage. Cancer Prev Res (Phila).

[43] Feng T, Li L, Ling S (2015). Metformin enhances radiation response of ECa109 cells through activation of ATM and AMPK. Biomed Pharmacother.

[44] Samsuri NAB, Leech M, Marignol L (2017). Metformin and improved treatment outcomes in radiation therapy - A review. Cancer Treat Rev.

[45] Zilinyi R, Czompa A, Czegledi A (2018). The Cardioprotective Effect of Metformin in Doxorubicin-Induced Cardiotoxicity: The Role of Autophagy. Molecules.

[46] Tseng YT (2016). Cardioprotective effect of metformin against doxorubicin cardiotoxicity in rats. Anatol J Cardiol.

[47] Kelleni MT, Amin EF, Abdelrahman AM (2015). Effect of Metformin and Sitagliptin on Doxorubicin-Induced Cardiotoxicity in Rats: Impact of Oxidative Stress, Inflammation, and Apoptosis. Journal of Toxicology.

